# Hypertension control and care at Mulago Hospital ambulatory clinic, Kampala-Uganda

**DOI:** 10.1186/s13104-016-2293-y

**Published:** 2016-11-17

**Authors:** Isaac Ssinabulya, Yvonne Nabunnya, Brian Kiggundu, Charles Musoke, Michael Mungoma, James Kayima

**Affiliations:** 1 Makerere University College of Health Sciences, Kampala, Uganda; 2 Mulago National Referral Hospital, Kampala, Uganda

**Keywords:** Hypertension, Blood pressure control, Management, Tertiary clinic

## Abstract

**Background:**

Hypertension is as prevalent in many developing countries, as in the developed world and is the leading cause of cardiovascular morbidity and mortality in Africa. The control of hypertension in this resource limited setting is inadequate, a situation that translates into poorer outcomes in form of increasing incidences of stroke, heart failure, kidney failure and therefore early cardiovascular death.

**Methods:**

This was a chart review of all the patients seen during the months of September 2012 to February 2013. We determined the level of blood pressure control, basic investigations documented as well as the choice of hypertensive treatment among patients attending a hypertension clinic in a national referral hospital, Mulago.

**Results:**

Of the 741 patients whose charts were reviewed the median age was 60 years, Inter quartile range (IQR) was 51–71. Six hundred forty-two (86.6%) were females. Blood pressure (BP) control defined as BP lowering to at least 140/90 was seen in only 198 (26.7%) patients. Biophysical measurement documentation was very low especially for waist and hip circumference at 0.3%. Majority of patients, 476 (64.2%) had at least one documented investigation for the complications of hypertension. Only 103 (13.9%) had all investigations documented in their charts. The investigations included; complete blood count (CBC), urinalysis, renal function tests (RFTs), Chest X-Ray (CXR), echocardiography (Echo) and electrocardiography (ECG). The commonly documented investigations were RFTs (45.5%), ECG (45.2%) and Echo (44.2%). The commonly prescribed anti hypertensive medications were; Angiotensin receptor blockers (ARBs)/Angiotensin converting enzyme inhibitors (ACEI) (72.74%), calcium channel blockers (72.3%) and thiazide diuretics (68.6%). Majority of patients were receiving three anti hypertensive medications 313 (42.2%), with 149 (43.6%) of these, on an ACEI/ARB, a calcium channel blocker and a thiazide diuretic.

**Conclusion:**

Blood pressure control is suboptimal in a tertiary clinic setting at Mulago hospital and documentation of investigations is inadequate. ARB/ACEI, Calcium channel blockers and thiazide diuretics were the commonly prescribed anti hypertensive medications. There is a great need to investigate for renal and cardiac complications as well as exploring reasons for inadequate blood pressure control and consider appropriate interventions to avert bad outcomes.

## Background

Hypertension affects about one billion people and is estimated to cause 4.5% of current global disease burden [1, 2]. There is a strong relationship between blood pressure control and cardiovascular mortality and morbidity [3]. Hypertension is the leading cause of cardiovascular morbidity and mortality in Africa where it affects young and active adults [4, 5]. The control of hypertension in this resource limited setting is likely to be inadequate, a situation that translates into poorer outcomes in the form of increasing incidences of stroke, heart failure, kidney failure and therefore cardiovascular death [6–8].

There is evidence that the diagnosis and control of hypertension is crucial in decreasing disability and mortality [9]. A meta analysis done by Lewington et al. demonstrated that BP reduction of 20/10 is associated with more than a twofold reduction in stroke death rate, and with twofold differences in the death rates from ischemic heart disease and from other vascular causes. [10].

Blood pressure elevation is usually a multifactorial condition, and therefore it is almost impossible to normalize pressure by targeting only one mechanism. In addition, drug therapy directed at any one component routinely evokes counter regulatory responses that reduce the magnitude of response. Clinical trials have documented that achieving BP targets is usually not possible with a single agent. In the Antihypertensive and Lipid-Lowering Treatment to Prevent Heart Attack Trial [11], only 26% of patients achieved goal BP with monotherapy. In contrast, combined therapy has been shown to improve control of blood pressure in an increased number of patients [12]. In addition to the improved efficacy of combination therapy, this strategy may have fewer side effects.

Blood pressure targets and choice of drugs are dependent on co-morbidity or presence of any target organ damage and so in the routine screening for patients with hypertension it is pertinent that some tests are done [13, 14].

There has been documented poor BP control among community studies [15]. However there have not been many studies that have evaluated clinic blood pressure control [16]. The utility of studying clinic based control lies in having the opportunity to work up patients for secondary causes and plan interventions.

In an attempt to improve patient care in our hypertension clinics, we determined the level of blood pressure control, basic investigations documented and the choice of hypertensive treatment among patients attending the tertiary hypertension clinic Mulago national referral hospital.

## Methods

### Study design and setting

This was a chart review of all the patients seen through the months of September 2012 to February 2013.

The study was conducted at the hypertension clinic in the medical out patients’ department of Mulago National referral hospital. The hospital is located in Uganda’s capital—Kampala and doubles as the teaching hospital for Makerere University College of health sciences. This clinic runs every Monday except on public holidays. The clinic is run by physicians, medical doctors and nurses plus support from the laboratory and pharmacy. About 80–100 patients are reviewed on a given clinic day.

### Data collection

We used the records book at the hypertension clinic to get a list of all the patients that attended the clinic through the months of September 2012 to February 2013, their files were retrieved and data was abstracted using a pretested tool. The team that collected data was trained before data was abstracted. The data abstraction form was piloted and adjusted to get the required information. The data collected included: socio-demographics [patient sex, and age], co-morbidities including diabetes mellitus, obesity, chronic kidney disease, stroke and clinical characteristics such as blood pressure, weight, height, and waist–hip circumference.

The investigations included: Renal function tests (RFTs), electrolytes, urinalysis, echocardiography, electrocardiogram (ECG) and Chest X-ray.

We also obtained information on the type of drugs prescribed to the patient.

### Operational definitions

An individual was classified to have sub-optimal pressure control if his/her blood pressure measurement was ≥140/90 mmHg on the last clinic visit. A missed appointment was defined as coming back to the clinic more than one week after a scheduled appointment.

### Data analysis

Data were collected manually and captured into Epi-data before it was transferred to STATA version 12 for analysis. Categorical variable are expressed as percentages and continuous data as median with their corresponding inter quartile range. Logistic regression was done to determine factors associated with blood pressure control. Statistical significance was set at p < 0.05.

### Ethical consideration

This research project was approved by the research and ethics committee of Mulago national referral hospital.

## Results

Of the 741 charts reviewed; 642 (86.6%) belonged to female subjects; the median age was 60 years Inter quartile range IQR (51–71). More than two-thirds 548 (74%) of patients were 50 years and above.

### Blood pressure less than 140/90 mmHg

Blood pressure control as defined as at least 140/90 was seen in only 198 (26.7%) of the patients whose charts were reviewed. Blood pressure control was better among female patients. Age was comparable among patients with good blood pressure control and those not controlled (Table 3).

### Co-morbidity

Ninety patients (12.2%) had documented co-morbid conditions. Stroke was in 14 (1.9%), Human immunodeficiency virus (HIV) infection was documented among 17 (2.3%), diabetes 11 (1.5%), asthma 11 (1.5%), arthritis 8 (1.1%), dyslipidemia 4 (0.5%) and benign prostatic hypertrophy in 4 (0.5%). Other conditions documented at very low frequency were renal disease, deep venous thrombosis, obstructive pulmonary disease, hyperthyroidism and peptic ulcer disease.

### Biophysical measurement

The proportion of patients with biophysical measurement was very low especially for waist–hip circumference (Table 1). Weight was documented among 266 (34%) patients, height in 169 (22.5%) patients, while waist–hip circumference were documented among (0.3%) patients whose charts were reviewed.

**Table 1:** Patient characteristics

Variable	Female n (%)	Male n (%)	Total n (%)
Age categories (years)			
<40	36 (5.8)	5 (5.2)	41 (5.7)
40–49	111 (17.9)	15 (15.6)	126 (17.6)
50–59	172 (27.8)	20 (20.8)	192 (26.9)
60–69	141 (22.8)	18 (18.7)	159 (22.2)
70–79	121 (19.6)	25 (26.0)	146 (20.4)
80 and above	38 (6.1)	13 (13.5)	51 (7.1)
Biophysical measurements done			
Weight	234 (36.5)	30 (30.3)	264 (35.6)
Height	148 (22.7)	20 (20.2)	168 (22.7)
WHR	2 (0.3)	0	2 (0.3)
Investigations done			
Complete blood count	231 (36.0)	35 (35.4)	266 (35.9)
Urinalysis	209 (32.6)	34 (34.3)	243 (32.8)
Renal function tests	292 (45.5)	47 (47.5)	339 (45.8)
Chest X-ray	114 (17.8)	18 (18.2)	132 (17.8)
Echo	284 (44.2)	48 (48.5)	332 (44.8)
ECG	290 (45.2)	51 (51.5)	341 (46.0)
Medication			
ACEi or ARB	464 (72.27)	75 (75.76)	539 (72.74)
Beta blocker	345 (53.7)	42 (42.4)	387 (52.2)
Calcium channel blocker	454 (70.7)	82 (82.8)	536 (72.3)
Thiazide diuretic	438 (68.2)	70 (70.7)	508 (68.6)
Centrally acting vasodilator	29 (4.5)	7 (7.1)	36 (4.9)
Potassium sparing	21 (3.3)	3 (3.0)	24 (3.2)
Furosemide	26 (4.1)	13 (13.1)	39 (5.3)
Co-morbidity	23 (23.3)	67 (10.4)	90 (12.2)
Blood pressure control	182 (28.4)	16 (16.2)	198 (26.7)
Missed appointment	301 (47.9)	45 (46.4)	346 (47.7)

*ARBs* Angiotensin receptor blockers, *ACEi* Angiotensin converting enzyme inhibitor

### Documented investigations

While the majority of patients had at least one documented investigation 476 (64.2%), only 103 (13.9%) had all the expected investigations documented in their charts. The expected investigations included CBC, urinalysis, renal function test, chest X-ray, echocardiogram and electrocardiography. The commonly documented investigations included RFTs (45.5%), ECG (45.2%) and Echo (44.2%) (Table 1).

### Medication

Several classes of anti hypertensive medications were used (Table 1). The most commonly prescribed medications were angiotensin receptor blockers (ARBs)/angiotensin converting enzyme inhibitors (ACEI) (72.74%), calcium channel blockers (72.3%), thiazide diuretics (68.6%) and beta blockers (52.2%). The least prescribed drugs were the centrally acting vasodilators and potassium sparing diuretics which were prescribed among 4.9 and 3.2% respectively. The use of a beta blocker, ACEi, calcium channel blocker or a thiazide was associated with poor blood pressure control (Table 3).

Majority of patients were receiving three anti hypertensive medications 313 (42.2%), with 149 (47.6%) of these on an ACEI/ARB, a calcium channel blocker and a thiazide (Table 2).

**Table 2:** Type of drugs used

	n (%)
One drug (5.8% n = 43)	
ARBs/ACEi	10 (23.3)
Calcium channel blocker	10 (23.3)
Diuretics	5 (11.6)
Beta blocker	15 (34.9)
Centrally acting vasodilator	1 (2.3)
Potassium sparing diuretic	2 (4.6)
Two drugs (32.8% n = 243)	
ARBs/ACEi and beta blocker	33 (13.6)
ARBs/ACEi and calcium channel blocker	66 (27.2)
ARBs/ACEi and thiazide diuretic	37 (15.2)
Beta blocker and calcium channel blocker	16 (6.6)
Beta blocker and thiazide diuretic	37 (15.2)
Others	6 (2.5)
Three drugs (42.2% n = 313)	
ARBs/ACEi, beta blocker and calcium channel blocker	53 (16.9)
ARBs/ACEi, beta blocker and thiazide diuretic	42 (13.4)
ARBs/ACEi calcium channel blocker and thiazide diuretic	149 (47.6)
Beta blocker, calcium channel blocker and thiazide diuretic	55 (17.6)
Others	14 (4.5)
Four drugs (16.5 n = 122)	
ARBs/ACEi, beta blocker, calcium channel blocker and thiazide diuretic	102 (83.6)
ARBs/ACEi, calcium channel blocker thiazide diuretic and centrally acting vasodilator	6 (4.9)
Others	14 (11.5)
Five drugs (1.9% n = 14)	
ARBs/ACEi, beta blocker, calcium channel blocker, thiazide diuretic and centrally acting vasodilator	5 (35.7)
ARBs/ACEi, beta blocker, calcium channel blocker, thiazide diuretic and potassium sparing diuretic	8 (57.1)
ARBs/ACEi, beta blocker, calcium channel blocker, centrally acting vasodilator and potassium sparing diuretic	1 (7.1)

*ARBs* angiotensin receptor blockers, *ACEi* angiotensin converting enzyme inhibitor

Blood pressure control varied across number of anti-hypertensive drugs used and was worse among patients taking 3 and 4 drugs; odds ratio (95% confidence interval) 0.32 (0.16–0.62) and 0.17 (0.08–0.37) respectively compared to monotherapy (Table 3).

**Table 3:** Factors associated with blood pressure control

Variable	Controlled blood pressure	Uncontrolled blood pressure	Odds ratio	p value	Adjusted odds ratio	p value
Age	61 (51–71)	59 (50–70)	1.01 (1.00–1.02)	0.195		
Gender—female	182 (91.9)	460 (84.7)	2.05 (1.17–3.60)	0.012	1.85 (1.03–3.34)	0.04
ARBs	78 (39.4)	232 (42.7)	0.87 (0.63–1.21)	0.416		
ACEi	55 (27.8)	201 (37.0)	0.65 (0.46–0.93)	0.02	0.53 (0.36–0.78)	0.001
Beta blockers	94 (47.5)	293 (54.0)	0.77 (0.56–0.83)	0.118	0.62 (0.44–0.88)	0.008
Calcium channel blockers	127 (64.1)	409 (75.3)	0.58 (0.41–0.83)	0.003	0.54 (0.37–0.78)	0.002
Centrally acting	5 (2.53)	31 (5.71)	0.43 (0.16–1.12)	0.083	0.36 (0.13–0.98)	0.046
Thiazide diuretic	120 (60.6)	388 (71.5)	0.61 (0.44–0.86)	0.005	0.54 (0.38–0.78)	0.001
Potassium sparing	9 (4.55)	15 (2.76)	1.67 (0.72–3.89)	0.23		
Number of anti-hypertensive drugs						
One	20 (10.1)	22 (4.1)	[1]			
Two	82 (41.4)	154 (28.4)	0.59 (0.30–1.14)	0.113		
Three	69 (34.9)	239 (44.0)	0.32 (0.16–0.62)	0.001		
Four	18 (9.1)	116 (21.4)	0.17 (0.08–0.37)	<0.001		
Five	5 (2.5)	9 (1.7)	0.61 (0.18–2.13)	0.44		
Having all investigations	24 (12.1)	79 (14.5)	0.81 (0.50–1.32)	0.399		
Having any comorbidity	25 (12.6)	65 (12.0)	1.06 (0.65–1.74)	0.809		
Keeping appointment	94 (48.2)	252 (47.5)	1.03 (0.74–1.43)	0.858		

*ARBs* angiotensin receptor blockers, *ACEi* angiotensin converting enzyme inhibitor

Other medications used included cardiac aspirin (23.4%), lipid lowering drugs (2.8%) and furosemide (5.3%).

### Missed appointments

Almost half of the patients 348 (47.7%) did not keep their appointments. There was no difference in blood pressure control between those that kept appointments and those that did not keep appointments OR 1.03 95% CI (0.74–1.43) p = 0.858.

## Discussion

Hypertension contributes to a high burden of disease and increased outpatient attendance for non communicable diseases. In Uganda specifically at the national referral hospital, the hypertension clinic is one of the busiest clinics with 80–100 patients reviewed each clinic day. Management of hypertension is aimed at controlling blood pressure to avert damage to end organs and thus improve quality of life for individuals with hypertension. In this study we found blood pressure control (as defined by a BP < 140/90 mmHg) at 26.7% which is inadequate in a country that has a high burden of hypertension [17, 18].

In this clinic, there were older people than the young, 49.7% of patients were 60 years old and above with only 5.7% below 40 years of age. This can be partly because in the young cause of hypertension is mainly renal disease (secondary hypertension) and these patients are reviewed in the renal clinic which independently runs on a different clinic day [19].

Hypertension control was comparable across age groups but this is different from what has been revealed from community studies that found better control among patients less than 50 years [20]. Borzecki et al. also found better blood pressure control among young adults but also noted that patients above 60 years were being treated less aggressively with fewer medications [21]. In our study, the number of drugs used across age groups wasn’t so varied.

Our finding illustrates a poor control compared to what Musinguzi et al. found in a community study, blood pressure control among those on treatment was achieved by 52 (35.9%) [17]. The possibility of white coat effect cannot be excluded, patients in the communities could be more relaxed compared to those in ambulatory clinics who might be anxious because of the long lines and long waiting times. There is also the fact that this population was highly selected and those feeling bad (symptomatic) are more likely to attend the hypertension clinic.

Documentation of biophysical measurements and investigations was exceedingly poor in this hypertension clinic (Table 1). It is not easy from this to ascertain whether this low documentation is because these parameters were not done in the first place. Documentation enables the reviewing physician to make appropriate decisions for a patient such as choice of drugs, management of side effects and co-morbidity, request additional tests as well as make appropriate referrals whenever needed. A simple measurement like weight can be crucial in monitoring patients with hypertension as this is crucial in the control and prevention of complications.

Urinalysis to check for micro-albuminuria can easily be done to screen for renal disease and plan management. Our study reveals that only a third of the patients (32.8%) had a urinalysis done. According to a study done by Nabbaale et al. in the same settings, 39.5% newly diagnosed hypertensive patients were found to have microalbuminuria [22]. This implies that a sizable proportion of our hypertensive patients can be reviewed for years with undetected renal disease until the disease has progressed to a level which is expensive to manage. The low documentation for biophysical measurements and investigations clearly calls for protocol guided care in our clinic setting to guide patient management.

Majority of our patients were taking more than one drug for their blood pressure control, with 42.2% taking three drugs. Despite having several patients on more than one drug we still had poor BP control in this clinic setting. According to the Eighth Joint National Committee (JNC 8) [23], the recommended first line treatment for blacks with hypertension is a thiazide diuretic or calcium channel blocker. ACEIs have been found to be less effective compared with the CCB in reducing BP in black individuals [24]. This is not necessarily the trend in our clinic, ACEI/ARBs are the most commonly prescribed drugs and whether this partly explains the lower rates of control needs further evaluation.

Black patients with hypertension and renal disease are recommended to take ACEI or ARBs according to the African American Study of Kidney Disease and Hypertension (AASK) [25]. Patients with CKD and hypertension usually require more than 1 drug to achieve goal BP and therefore an ACEI or ARB is used either as initial therapy or as second-line therapy in addition to a diuretic or CCB in black patients with CKD. However in our patient population little is known about CKD status and so use of ACEI or ARB is not from an informed point of view. This emphasizes the need to screen patients and also study this further in our patient population.

We found that majority of patients on monotherapy were taking beta blockers, this is not in line with the guidelines. Beta-blockers are not recommended for the initial treatment of hypertension because their use resulted in a higher rate of cardiovascular death, myocardial infarction, or stroke [26]. Beta-blockers are readily available and cheap in Uganda, this could explain their common use.

This finding of low blood pressure control prompts us to consider other modalities that can contribute to better blood pressure control. There have been studies that have shown that renal denervation may be a cost effective approach compared to medical management in management of resistant hypertension [27, 28]. The use of renal denervation is still controversial with demonstrated no benefit from the Simplicity 3 trial [29], however some scholars believe that renal denervation done by experienced hands and good patient selection can be beneficial. We cannot conclude though that all patients that had poor control have resistant hypertension but this is an aspect that needs consideration knowing that some patients could have resistant hypertension. In Africa, as we advance in catheter based interventions by our physicians; nerve denervation could be a welcome solution among patients with resistant hypertension.

This was a retrospective chart review study; we could not ascertain why control is low however we describe the magnitude of the problem and highlight key gaps in documentation. we cannot conclude that tests were not done since we went by what was documented but this clearly demonstrate that we have room to improve and make the care for our patients better.

Evaluation for a hypertensive patient ought to be comprehensive to identify risk factors, co-morbidity as well as stratify patients to risk categories and plan appropriate treatment. In a clinic setting there is opportunity to make management of hypertension better and consequently avert the bad outcomes that are associated with poor blood pressure control.

## Conclusions

Blood pressure control is suboptimal in a clinic setting at Mulago hospital and documentation of basic investigations is inadequate. ARB/ACEI, Calcium channel blockers and thiazide diuretics were the commonly prescribed anti hypertensive medications. There is an urgent need to explore reasons for suboptimal control and institute measures to avert cardiovascular complications that are associated with poor blood pressure control.

Evaluation for a hypertensive patient ought to be comprehensive to identify risk factors, co-morbidity as well as stratify patients to risk categories and plan appropriate treatment. Protocols have to be put in place and implemented for better patient care. In a clinic setting there is opportunity to make management of hypertension better and consequently avert the bad outcomes that are associated with poor blood pressure control.

## Abbreviations

AASK: African American Study of Kidney Disease and Hypertension
ACEI: angiotensin converting enzyme inhibitors
ALLHAT: antihypertensive and lipid-lowering treatment to prevent heart attack trial
ARB: angiotensin receptor blockers
CCB: calcium channel blocker
CKD: chronic kidney disease
CXR: chest X-ray
ECG: electrocardiogram
JNC: joint national committee
